# Leveling up fun: learning progress, expectations, and success influence enjoyment in video games

**DOI:** 10.1038/s41598-025-14628-2

**Published:** 2025-10-01

**Authors:** Franziska Brändle, Charley M. Wu, Eric Schulz

**Affiliations:** 1 University of Oxford, Oxford, UK; 2 Human and Machine Cognition Lab, University of Tübingen, Tübingen, Germany; 3 Max Planck Institute for Biological Cybernetics, Tübingen, Germany; 4 Centre for Cognitive Science, Institute of Psychology, Technical University of Darmstadt, Darmstadt, Germany; 5 Hessian.AI, Darmstadt, Germany; 6 Helmholtz Institute for Human-Centered AI, Helmholtz Center Munich, Munich, Germany

**Keywords:** Fun, Enjoyment, Engagement, Curiosity, Learning progress, Psychology, Human behaviour

## Abstract

What factors influence how much fun people have when engaging in inherently enjoyable tasks? Several theories predict that people will have the most fun in environments of intermediate difficulty because these environments usually offer the most progress in learning about the world. Past studies have frequently focused on simple experimental paradigms in which learning was still instrumental for later tasks. Here, we put these theories to a test in three large and realistic video game data sets: a puzzle game (with 7,994 levels and 376,341 votes), a racing game (138,662 levels and 614,770 votes), and a platformer game (115,032 levels and 795,313 votes). As predicted, people preferred levels of intermediate difficulty in all games. Yet, additional factors influencing people’s enjoyment also emerged: players preferred levels that matched closely with their prior expectations of difficulty and were also motivated by success. We further confirmed these factors in two precisely controlled experiments. Taken together, these results advance our understanding of the dynamics of fun in realistic environments and emphasize the importance of using both realistic, game-like environments and highly controlled experiments to refine theories of human learning and decision-making.

## Introduction

People engage in many activities not only for material rewards or instrumental benefits but simply for the fun and enjoyment they offer. From playing a game, or taking in a movie, to making music, there are countless activities where behavior appears to be intrinsically motivated absent of instrumental benefits. With the high number of enjoyable options available at any time, it becomes crucial to understand which activities people prefer and continue to participate in purely for fun. What are the features influencing whether or not an ongoing activity is experienced as intrinsically rewarding and thus predicting continued engagement, especially in rich and realistic scenarios?

Intrinsic motivation has often been linked to an optimal level of task difficulty. Multiple theories — based on factors such as novelty^1^, achievement^2^, or challenge^3^ — propose that intrinsic motivation peaks with tasks of intermediate difficulty (or knowledge), resulting in an inverted-U relationship between the two variables^4^. This phenomenon has been observed across various research domains, including curiosity^5–7^, boredom^8^, flow^9^, development^10^ and game design^11^. Although these theories are grounded in different conceptual frameworks, recent work has argued that many of them can be unified under the principle of resource-efficient knowledge maximization^4^: in many natural environments, preferring tasks of intermediate difficulty constitutes an optimal strategy for acquiring knowledge about the world. When a task is too easy, people will not learn much, since they will have already possessed the necessary knowledge or skills to master it: for example, teaching an adult how to tie their shoes, or a skilled chess player how to move their pawns. On the other hand, when a task is too difficult, people might struggle to achieve any degree of traction, since they may fail miserably without any real lessons being learned: for example, teaching a 1-year-old to tie their shoes or a novice chess player how to play the Ruy Lopez opening.

**Fig. 1:**
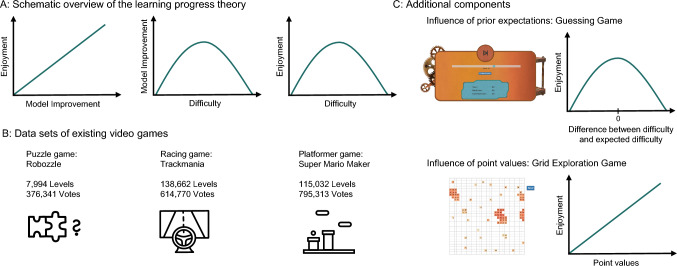
Visualization of the tested theory and experimental setup. (**A**) The learning progress theory. According to the theory, enjoyment increases with model improvement. As shown in multiple studies and our simulations, model improvement is highest in environments of intermediate difficulty. Therefore, enjoyment is predicted to be highest in environments of intermediate difficulty. (**B**) Details on the three game data sets. We chose three pre-existing games of different genres, all including a large set of levels and player votes. (**C**) Overview of our additional experimental setups. First, we investigated the additional component of difficulty-expectation disparity in a guessing game, where people had to guess numbers generated by different machines (for an enlarged version of the paradigm, refer to Fig. 3a). Second, we investigated the additional component of success in a grid exploration game, where people could open numerical tiles on a large grid.

One of these theories that formally described “fun” — or more generally intrinsic reward — is consequently based on an informational goal of learning progress^12–14^. A common technique used in robotics and machine learning is to assign intrinsic rewards to the active creation or discovery of surprising experiences, which allow intelligent agents to improve the predictions of their model of the world^12^. According to this approach, a subjective measure of “fun” or intrinsic reward can be defined as the degree to which the world model improves^15,16^. This again produces an inverted U-shaped relationship between difficulty and intrinsic reward, with intrinsic reward being the highest in situations of intermediate difficulty (i.e. not too easy and not too hard) where learning progress is maximal (Fig. 1a). From a normative perspective, robots learn faster when maximizing learning progress^17,18^ and machine learning models using stochastic gradient-descent learn best when they are correct roughly 85% of the time^19^. Descriptively, learning progress has been linked to influential accounts of curiosity^20,21^ and development^1^, with distinct neural responses encoding the anticipation of obtaining information compared to anticipations of reward^22–24^.

However, empirical evidence for human engagement being driven by learning progress has only recently emerged, often using free choice paradigms where task difficulty is related to learning outcomes^25^, engagement^26,27^, and subjective ratings^28^. A recent pre-registered study^29^ provided additional support that learning progress rather than difficulty is a main driver of enjoyment. Using a “dynamic difficulty adjustment” system, learning progress was deliberately suppressed, with the outcome that the researchers failed to find a relationship between the difficulty-skill balance and enjoyment^29^.

In his seminal paper, Schmidhuber argues that making learning progress results in “fun”^30^. However, “fun” lacks a consistent definition in psychological literature and refers to a complex and subjective phenomenon^31,32^ . Here, we will use the broader term “enjoyment” to describe the positive affect people experience when engaging in inherently “fun” tasks, such as games. While we believe that our paradigms capture the essence of fun and enjoyment, we adopt the term “intrinsic reward” to describe participants’ positive experience elicited by learning progress — as proposed by Oudeyer et al.^16^ — to avoid unwarranted generalization. The term originates from the intrinsic motivation literature, which defines intrinsically motivated activities as those “for which there is no apparent reward except the activity itself” (REF^3^, p. 23). Traditionally, intrinsic motivation and rewards have been measured through behavioral measures, such as engagement^3^ and choice behavior^25^, or through self-report measures^7^. In our work, we use both engagement and players’ level ratings to gain a more comprehensive understanding of learning progress.

While past studies have focused on simple experimental paradigms in which learning was still instrumental for later tasks, here we are interested in settings such as video games or other enjoyable diversions, where people play purely for fun or pleasure and can decide to quit at any moment^33^. In these settings, people can still gather knowledge about the structure or difficulty of the task, but instrumental learning is not the main focus of the activity^6,15,34^. In these inherently enjoyable scenarios, optimizing knowledge accumulation may not be the only motivational driver, since the link to a normative basis for improving future outcomes is made more tenuous. Thus, it is an open question whether inverted-U theories can provide a good description of behavior in more real-world and inherently fun contexts.

### Goals and scope

**Fig. 2:**
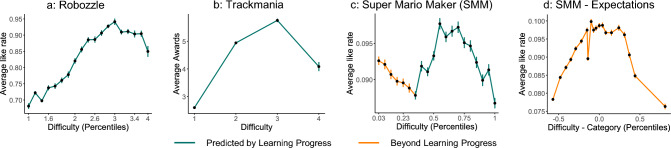
Rating behavior in game data sets. (**a**) People’s ratings of different levels of Robozzle. (**b**) People’s ratings of different levels of Trackmania. (**c**) People’s ratings of different levels of Super Mario Maker. The orange part indicates that players also liked simple levels, on top of levels with intermediate difficulty. (**d**) People’s ratings of levels in relation to their expectations of difficulty and our difficulty calculation (success ratio). We plot the difference between the difficulty calculation — based on attempts and clears of the levels — and expected difficulty — given by categories assigned by the creators — in Super Mario Maker. Error bars indicate the standard error of the mean.

To answer this question, we used multiple large-scale game data sets, as well as two online experiments. Within three large-scale video game data sets (“Robozzle”, “Trackmania”, and “Super Mario Maker”; Fig. 1b), we used subjective ratings as an indicator of intrinsic reward, finding an inverted U-shaped relationship between difficulty and people’s enjoyment (Fig. 2). However, other important factors also emerged. In particular, the Super Mario Maker data set revealed that players’ ratings were also influenced by i) a “difficulty-expectation disparity” based on the mismatch between prior expectations about the difficulty of a level and our calculations of the underlying difficulty and ii) “success” from completing levels with low difficulty.

**Fig. 3:**
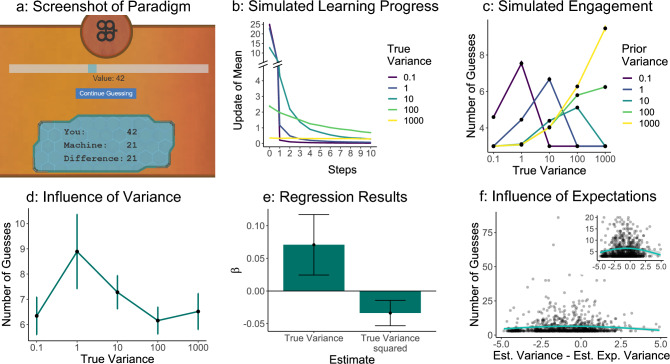
Overview of guessing game experiment. (**a**) Game design. Participants interacted with machines producing numbers from Gaussian distributions by adjusting a slider to guess the number the machine would produce next. After each guess, they could decide whether to stay with the current machine or move on to the next one. (**b**) Simulated learning progress with a Kalman filter. The trajectory of the update of the mean depends on the true underlying variance of the Gaussian distribution. The smaller variances make large updates only during the first step, while the high variances make only small updates directly from the first step onwards. Only intermediate variances continue to make larger updates. (**c**) Simulated engagement. The simulation samples from the current distribution until the update of the mean lies below a threshold — here set to 0.5 (for other values, see SI). The peak of the inverted-U shapes — the true variance the simulation is interacting with the longest — depends on the prior variance. (**d**) Behavioral results. Data from 98 participants showed that they liked to interact most with machines with variance 1. (**e**) Mixed-effects regression analysis. The significant negative squared effect of the variance accounts for the inverted U-shape seen in the human engagement behavior. (**f**) Influence of the difficulty-expectation disparity on human behavior. The difference between the estimated variance — calculated based on the samples participants have seen in the current distribution — and the estimated expected variance — calculated based on the machines participants have seen so far — shows an inverted-U relationship to the number of guesses with a peak close to 0. This indicates that participants preferred to interact with variances that lie close to their expected variance. The inset plot displays a zoomed-in version of the data. Error bars (in d and e) indicate the standard error of the mean.

**Fig. 4:**
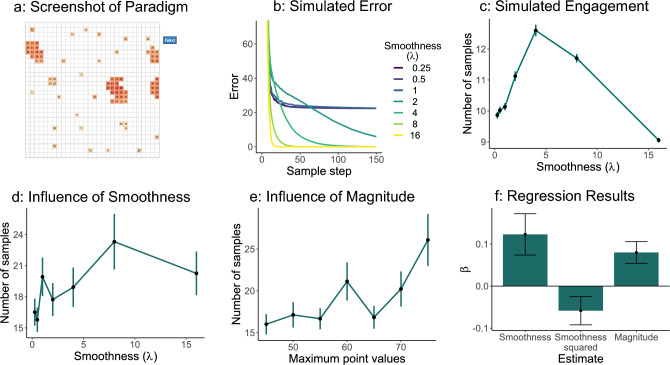
Overview grid exploration game. (**a**) Game design: Participants explored tiles of a grid produced by an underlying Gaussian Process. We manipulated the smoothness and the point values of the grids. After each sample, participants could decide to open another tile or go on to the next grid. (**b**) Simulated error over time with a Gaussian Process. The trajectory of the error — calculated as the mean squared error between the true values and the predicted values of the grid — is dependent on the smoothness of the grid, defined by the underlying lengthscale parameter ($\lambda$). Smaller values plateau quickly, while larger values quickly reach an error of 0. Only intermediate values continue reducing their error over a longer time frame. (**c**) Simulated engagement. The simulation samples new tiles until the error lies below a threshold — here set to 0.5 (for other values, see SI). We see an inverted U-shape relationship between the smoothness — defined by the lengthscale parameter ($\lambda$) — and the number of samples. (**d**) Behavioral results based on smoothness. We gathered data from 44 participants. They interacted the most with grids with a $\lambda$ value of 8. (**e**) Behavioral results based on the magnitude of point values. Values are summarized in bins of 5. Participants interacted the most with grids that had high point values. (**f**) Mixed-effects regression analysis. The significant negative squared effect of smoothness accounts for the inverted U-shape seen in the behavioral plot. The linear effect of magnitude showed that participants engaged most in environments with higher point values. Error bars indicate the standard error of the mean.

Having established that the inverted U-shape relationship between difficulty and intrinsic motivation, along with the difficulty-expectation disparity and success, influences player’s ratings in complex and realistic video games, we conducted two controlled experiments (Fig. 1c) to further corroborate our findings — measured through player engagement. Here, we test the theory of intrinsic reward as learning progress^15,16,35,36^ to inform our simulations and to compare them with human behaviour. In Experiment 1 (Fig. 3), we used a simple guessing game, where participants guessed numbers from Gaussian distributions with different levels of variance defining the difficulty. In both simulations and participant data, we found that learning progress and the difficulty-expectation disparity stemming from a mismatch between expectations about difficulty and true difficulty influenced players’ engagement with each machine. In Experiment 2 (Fig. 4), we developed an open-ended exploration paradigm, in which participants explored different grids containing hidden points with varying degrees of spatial correlation. Through both simulations and participant data, we found that in tandem with increased engagement in levels with higher learning progress, participants also engaged more with landscapes with higher underlying point values. Taken together, these results advance our understanding of higher-order concepts in human motivation and intrinsic reward, by showing that multiple factors influence people’s ratings and engagement behavior in inherently enjoyable, realistic, and rich environments.

## Game datasets

To investigate the relationship between intrinsic motivation and difficulty in inherently non-instrumental and enjoyable settings, we decided to use video games as a testbed. Games have a long history in psychology^37^. Video games bring the additional benefit that they provide a lot of data, as they are by design enjoyable and therefore attract more participants and generate more data per participant^38^. Video games are also more complex than traditional psychological paradigms, which brings them closer to the complexity of real-world environments^33^.

In our analyses, we focused on three large-scale data sets of the popular video games Robozzle, Trackmania, and Super Mario Maker. All three games have in common that players can create and contribute levels, which other players can subsequently play and rate. These games, therefore, offer a great diversity in the complexity and difficulty of many different levels. We compared the influence of different levels of difficulty on enjoyment within each game. We expected to see an inverted U-shape between difficulty and enjoyment for each game — as predicted by multiple theories of intrinsic motivation. As the three data sets did not share the same data structures, we approximated enjoyment and difficulty measures for each game individually, based on the available data.

### Robozzle — Results

Robozzle is a puzzle game in which players need to write small, often recursive, programs with the help of building blocks to steer a spaceship to its goal. The data set consisted (after cleaning, see SI) of 7,994 levels and 376,341 votes. Players could rate the difficulty of each level between 1 and 5. For our analysis, we used the average rating for each level, which was displayed on the website as a value between 1 (very easy) and 5 (very hard) in steps of 0.01. Players could also up- and downvote levels, which we transformed into an enjoyment measure by dividing the upvotes by the total number of votes to get a like-ratio. Analyzing votes, we found that players seemed to prefer intermediate difficult levels over very easy or hard levels (see Fig. 2a). Additionally, we ran a regression analysis and found a significant negative quadratic effect of difficulty, indicating an inverted U-shape as predicted by the learning progress theory (linear: $\beta =0.09$, $z=44.37$, $p<.001$, quadratic: $\beta =-0.04$, $z=-22.98$, $p<.001$). Due to the size of the data set, standard model comparison methods were not appropriate. Instead, we looked at the model fit of different polynomial regression models. We observed that adding a quadratic term improved the model fit meaningfully, while adding a cubic term did not. For details on model comparison, refer to the SI. However, it is important to note that a difficulty measure based on players’ ratings instead of their performance might lead to a tautological situation in which players rate the levels they enjoyed the most as intermediate difficult^29^. We avoid this confounding factor in the two upcoming datasets.

### Trackmania — Results

Trackmania is a car racing game, in which players steer a racing car through different artificial-looking tracks. The data set consisted of 138,662 levels and 614,770 votes (here called awards). The difficulty of each level was given by the website as one of four categories: Beginner, Intermediate, Expert, and Lunatic. These difficulties were assigned by the creators of the levels. We mapped these difficulty categories to the values 1 - 4. Players could give awards to levels, of which we used the absolute number per level as an enjoyment approximation. Again, players seemed to like intermediate difficult levels more than very easy or hard levels (see Fig. 2b). In an additional regression analysis similar to the previous one, we again found a significant negative quadratic effect of difficulty as expected (linear: $\beta =1.10$, $z=16.62$, $p<.001$, quadratic: $\beta =-0.47$, $z=-10.63$, $p<.001$). We again observed that adding a quadratic term meaningfully improved the model fit, while adding a cubic term did not (see SI).

### Super Mario Maker — Results

Super Mario is a platformer video game series, in which players have to move the main character to a goal by mainly jumping, running, and avoiding enemies. In Super Mario Maker, players can create their own Super Mario-style levels and then upload their levels for other players to play and rate them. We analyzed the data of 115,032 player-designed levels and 795,313 votes, which were based on a total of 32,665,615 attempts. As we had more detailed data per level, we did not have to rely on players’ or creator’s difficulty ratings. Instead, we approximated the difficulty of each level as 1 minus the ratio of how many players who attempted a level were able to finish it. Players could rate levels with stars. We defined our enjoyment measure as the proportion of players attempting a level, who also gave a star to this particular level. As in our previous experiments, we found an inverted U-shaped relation between our difficulty and our enjoyment measure (Fig. 2c). Again, players seemed to like intermediate difficult levels the most. As in our previous regression analyses, we found a significant negative quadratic effect of difficulty, as predicted (linear: $\beta =0.001$, $z=3.62$, $p<.001$, quadratic: $\beta =-0.002$, $z=-8.63$, $p<.001$). However, a closer look at the data suggests that it does not follow a quadratic function and is better described by a third-degree polynomial (see Fig. 2c). Indeed, we found that adding a cubic term improved the model fit (see SI). Therefore, while we found an inverted-U shape relationship in all three data sets, the Super Mario Maker data set appeared to involve more than just a preference for stimuli with intermediate difficulty. When only looking at the lower difficulties, it looked like players preferred simpler levels over more difficult ones. There are multiple possible explanations for this phenomenon:

First, players might have been influenced by their prior expectations about the difficulty of a level and how well it matched the actual difficulty. In the Super Mario Maker data set, each level was placed into one of four difficulty categories by the creator. These categories did not always match our difficulty calculations — defined by the number of players managing to clear the level (see SI). We looked at the discrepancy between these two difficulty assessments, by relating the difference between the calculated difficulty and the expected difficulty — defined by the difficulty category — to the average like rate (for more details, see SI). We observed an inverted-U shape with the peak at around 0 (see Fig. 2d). Players preferred levels in which their expectations matched our calculated difficulty measure. To assess this in more detail, we ran an additional regression analysis, including the difference between the two difficulty measures, and found a significant negative quadratic effect of the difference (linear: $\beta =0.00$, $z=-8.29$, $p<.001$, quadratic: $\beta =-0.06$, $z=-40.12$, $p<.001$). As the linear effect is close to 0, the regression analysis confirmed that the peak of the inverted U-shape lies at a difference of 0 — where the expected difficulty matches the calculated difficulty measure.

A second possibility for the negative trend in the first half of the Super Mario Maker data set might be that players simply enjoy the feeling of success (e.g. solving a level or receiving higher rewards) on top of making progress in learning — thereby preferring the very easy over the slightly more difficult levels. The influence of rewards in video games on motivation has been shown in previous work^39^. However, with the data from the game data sets, we are not able to tease difficulty and a preference for success apart.

All of the above-mentioned data originated from three large-scale data sets of popular existing games. While this provided us with a large amount of data per level, we only had limited access to data per player. We were therefore not able to further study the additional hypotheses — i.e. the influence of the difficulty-expectation disparity or the influence of success. Instead, we decided to design two controlled and gamified experiments, in which we can collect all the necessary data, run more detailed analyses and precisely simulate learning progress. With the first experiment — a guessing game — we assessed the influence of difficulty on people’s engagement, including the relation of prior expectations of difficulty to the true difficulty. With the second experiment — a grid exploration game — we looked at the additional influence of success by letting players explore point values in grids.

## Experiment 1: A guessing game

To test the influence of learning progress on engagement in a simple setting, we developed a guessing game in which players guessed numbers from Gaussian distributions with different variances. This simple task has two main advantages over the above-analyzed game data sets: First, it allows us to implement a rational model and thereby derive more concrete predictions. Second, it minimizes the effect of the prior skill level of players, as the difficulty is defined by the randomness of the environment. While the task resembled classical psychological paradigms, we maintained the idea of using games as a research platform by giving players only a base pay and not compensating for performance. Additionally, we gave players the option to not engage with the task while still receiving their base compensation. Thereby, we still assessed enjoyment, rather than instrumental information gathering.

### Paradigm

This experiment was inspired by the work of Geana et al.^8^ In one of their experiments, participants guessed numbers generated by a virtual machine in one of three conditions — “Certain”, “Gaussian” and “Random” — while regularly reporting their boredom levels. Consistent with the learning progress theory, people’s boredom levels were lowest in the Gaussian condition, in which learning was actually possible.

We adapted the experiment by introducing five Gaussian conditions with different underlying variances, which manipulate the difficulty of correctly guessing a number. Participants interacted with the distributions by guessing which number the machine would produce next (see Fig. 3a). After submitting the number, we displayed the number the machine produced, as well as the difference between their guess and the machine’s number. After each guess (after a minimum of three guesses), people could either continue guessing with the current distribution or stop and go on to a new distribution (with a new mean and variance), until the experiment automatically ended after a certain time (for more details, refer to the methods section). Importantly, players knew that they would not receive any performance-dependent bonus and did not need to interact with the game, so everything they engaged in during the time was purely for their entertainment. Thereby, we strongly focused on the enjoyment aspect of the game. We measured how many guesses participants played with each machine as an approximation of how much they enjoyed interacting with it.

We predicted that, in this controlled environment, participants would act according to the theory of learning progress. Consistent with other inverted-U shaped theories, this would result in them spending most time with machines with intermediate variances, as they offer the most potential for learning. Machines with small variances would produce the same number repeatedly, while machines with big variances create numbers almost randomly. In both cases, participants would not make learning progress over a longer time which should prevent their enjoyment and consequently lead to participants changing to the next machine.

### Simulation

To model this behavior, we used a Kalman filter, which is a Bayesian agent that integrates observations optimally to generate an estimate of the current mean. Initially, we assumed a prior variance of 10 and a prior mean of 50. With each sample, the Kalman filter updates the estimates of the mean and variance according to the Kalman Gain, which depends on the relationship between the estimated variance and the true underlying variance (for more details, see SI). We believe that participants try to track the mean of the distribution, as choosing the mean would give them the best chance to be close to the next generated number. Here, we therefore define learning progress as the update of the mean at each time step. As can be seen in Figure 3b, the trajectory of learning progress differs based on the underlying variance. When simulating distributions with small variances (i.e. 0.1 or 1), the initial update at step 0 was large but is approaching 0 fast over the next few steps. When simulating distributions with high variances (i.e. 1000), there was almost no learning progress from the beginning onwards. When looking at intermediate variances (i.e. 10 or 100), the agent took an intermediate-sized step in the beginning but kept on making learning progress in the following steps. Which variances qualify as “small”, “big” or “intermediate” is, of course, dependent on the assumed prior variance, which, in this case, was set to 10.

We assumed that people engage with a machine as long as their learning progress per step lies above a certain threshold. To simulate the amount of engagement — the number of guesses until they turn to the next machine — we stop the simulations when the update of the mean lies below this threshold, here set to 0.5 for simplicity (for other threshold values, see SI). We force the simulation to guess at least three times, similar to the experiment. We compared the different points of stopping based on the true and the prior variance. Each setup displayed an inverted U-shape when plotting the true variance on the x-axis and the number of guesses on the y-axis. Furthermore, the peak of the inverted U-shape was determined by the prior variance — the lower the prior, the lower the peak of the curve (see Fig. 3c). This indicated that participants might like to interact with distributions with variances that are close to what they expect as prior variances.

### Results

We used the data of 98 participants collected via Amazon Mechanical Turk. Participants played on average with 14 machines (mean 13.68, SD 8.55), made on average 77 guesses in total (mean 77.35, SD 27.65) and 8 guesses per machine (mean 8.42, SD 7.74).

First, we checked whether the manipulation of the variance indeed manipulated the difficulty of guessing correctly. We found that, with increasing variance, the average difference between the guesses and the numbers produced by the machine increased ($\beta =5.79$, $t=33.03$, $p<.001$), while the number of correct guesses decreased ($r=-0.94$), as expected (for more details, see SI).

We examined whether participants’ guess updates diminished over time, signifying convergence in their guesses. This would indicate progress in guessing the numbers generated by the machines. We performed a regression analysis, using the scaled update — the absolute difference between the current and last guess — and included a random intercept for each participant. We found that the number of the current trial had a significant negative effect on the size of the current update ($\beta =-0.63$, $z=-5.80$, $p<.001$): players’ updates got smaller over time for each machine, indicating that they converged on a number to guess.

We assessed whether players acted as predicted by the learning progress hypothesis — and other inverted-U shaped theories — in this controlled environment by investigating the relationship between variance and engagement, measured by the number of guesses participants made with each machine. According to our simulations and the original theory, players should engage most with environments with intermediate variance. We first plotted the average number of interactions over variances and participants (see Fig. 3d). We saw that people showed an inverted U-shape relationship and engaged most with machines of variance 1. To confirm this result, we performed a negative binomial mixed-effects regression analysis including the variance and the squared variance as components while adding a random intercept for each participant. We found that the linear component had a significant positive effect ($\beta =0.07$, $z=2.99$, $p=.003$), while the squared component had a significant negative effect ($\beta =-0.03$, $z=-3.40$, $p<.001$), which indicated an inverse U-shape (see Fig. 3e). Hence, participants engaged most with intermediate variances.

Additionally, we wanted to test whether participants’ difficulty-expectation disparity — the mismatch between prior expectations of the current variance and the true variance — influenced their playing behavior, similar to what we had observed in the Super Mario Maker data set. We approximated players’ prior difficulty by considering the machines participants had encountered thus far in the experiment. To calculate the current expectation, we took the average of the observed estimated variances up to this point, weighted by the number of guesses for that round. Additionally, we calculated the estimated variance of the current machine based on all the samples the machine produced before stopping. We then looked at whether the difference between the estimated variance and the estimated expected variance influenced participants’ engagement (for details and different versions, see SI). Again, we found an inverted U-shape relationship between the difference and the number of guesses (see Fig. 3f). A negative binomial mixed-effects analysis supported this finding. While controlling for the number of machines played so far and including a random intercept for each participant, we found a significant negative effect of the scaled difference squared ($\beta =-0.06$, $z=-4.16$, $p<.001$), but no significant effect of the scaled linear component ($\beta =0.02$, $z=1.33$, $p=.185$), indicating that the peak of the inverted U-shape was not significantly different from 0. This indicates that participants engaged most with distributions with a variance close to their prior expected variance.

## Experiment 2: an exploration game

People seem to take learning progress and the difficulty-expectation disparity into account when deciding how long to engage with each distribution in the guessing game. However, as we saw in the Super Mario Maker data set, players might also be motivated by simply having a preference for success. We believe that players prefer to interact with environments that offer higher reward values, even if these are not cumulative and players are not instructed or compensated to do so. Ideally, we wanted to test the influence of this additional component in a second experiment, in which we could discriminate between the influence of difficulty and the influence of uncompensated rewards on players’ behavior. Moreover, we wanted to expand our initial observations on to another setting in which learning progress could be directly manipulated. Thus, we decided to use an additional paradigm for our second experiment — a grid exploration game. In this game — based on the grid search paradigm developed by Wu et al.^40–42^ — we could not only manipulate the difficulty of the environment but also its point values, independent of each other. Additionally, this setting was somewhat richer than the original guessing game, thereby further closing the gap between lab experiments and games.

### Paradigm

In this game, participants interacted with multiple grids by iteratively opening tiles and observing their point values (see Fig. 4a). At each time step (after a minimum of five tiles), they could decide whether they wanted to open another tile or if they wanted to go to a new grid until the experiment automatically ended. They were only instructed to explore the grids, and no specific goal was given. As before, players knew that they would not get any performance-dependent bonus, and did not need to interact with the task: they again were free to use the time as they liked. We approximated engagement by measuring how many tiles participants opened in each grid.

We manipulated the structure of the grids across two dimensions. We manipulated the difficulty of predicting the value of a tile by changing the smoothness (the spatial correlation) of the underlying landscape of values, defined by a lengthscale parameter $\lambda$. The smaller the lengthscale parameter, the rougher the landscape — the value of a tile gives almost no information about the values of its neighbouring tiles. The bigger the lengthscale, the smoother the landscape — the value of a tile gives a lot of information about the values of its neighbouring tiles. The second dimension is the value of points — some grids constituted of higher point values, some of lower point values.

We assumed that participants would prefer to interact with intermediate difficult environments, which corresponded to intermediate lengthscales in this paradigm, as these lead to the biggest potential for learning progress. We, therefore, expected to see an inverted U-shape relationship between the lengthscale parameter $\lambda$ and the engagement of participants. Additionally, we predicted that participants would engage more with grids that had a higher point value range. We assumed that participants enjoy the feeling of success, which we believe occurs when sampling high-value tiles. Thus, we expected to see a linear relationship between the maximum point values and participants’ engagement.

### Simulation

We first tested the learning progress hypothesis by conducting several simulations with a Bayesian learning model using Gaussian Processes. The learning curves of the Gaussian Process model can be calculated, providing insight into the predictability of each function^43^. Schulz et al. showed that these learning curves match human behavior. Additionally, manipulating the smoothness (defined by the lengthscale parameter $\lambda$) influences people’s perceived predictability of the functions^44^. For this simulation, we generated 1000 grids per lengthscale parameter. In each grid, we iteratively randomly sampled and opened tiles. After each tile, we let the model update its predictions of the values of all other tiles in the grid, based on the samples so far. We then compared this prediction to the ground truth of the grid to calculate the mean squared error. We took the mean over all grids for each lengthscale parameter and plotted the development of the error over time (see Fig. 4b) to assess the learning progress of the model for the different lengthscale values ($\lambda$). We found that for small $\lambda$ values (i.e. 0.25, 0.5, and 1), the model reduced the error very fast in the beginning, but then quickly plateaued. For large values (i.e. 8 and 16), the model also reduced the error fast but approached 0 very quickly. In both cases, the model did not show learning progress over a longer time. However, for intermediate values (2 and 4), the model learned over a longer period, which supports our hypothesis.

We simulated how long the model would engage with each grid. We set a threshold to the learning progress — the lower limit of what the model would consider as worth for continued engagement. In the current simulation, we again set the threshold to 0.5 for simplicity. However, the qualitative results — an inverted U-shape relationship between the lengthscale and number of samples — stay the same when changing the threshold (for examples of different thresholds, see SI). We then measured how many tiles the model would open before the absolute difference of the error between two steps trailed below the threshold while forcing the simulation to also open at least five tiles of each grid. We saw that, indeed, there exists an inverted U-shape relationship as expected (see Fig. 4c).

For the magnitude of uncompensated points, we did not run any simulations, as we expected a simple linear relationship between engagement and the point values of a grid.

### Results

To test whether people were influenced by the smoothness and the point values of the grids, we ran an experiment on Amazon Mechanical Turk with 44 participants. Participants interacted on average with 40 grids (mean 39.57, SD 37.16) and opened on average 36 tiles per grid (mean 36.09, SD 28.99). We assessed how much they engaged with each grid as a function of the lengthscale ($\lambda$) (see Fig. 4d) and magnitude of point values (see Fig. 4e). Participants liked to interact most with a lengthscale value of 8 confirming our hypothesis about the smoothness of the environment influencing engagement. While the downward trend of the second half of the inverted U-shape is driven by only one data point ($\lambda$ = 16), we believe that testing higher lengthscale values will not lead to any further insights, as the grids will become increasingly non-differentiable^45^. Participants also liked to interact most with the highest point values, confirming our hypothesis about the magnitude. We saw that especially environments with high point values positively influenced the number of tiles participants opened, similar to what we observed in the Super Mario Maker data set — players exhibited a linear relationship in the easier environments.

Additionally, we ran a negative binomial mixed-effects regression analysis using the scaled value of the lengthscale and the scaled value of the magnitude, including a random intercept for each participant, and found that the lengthscale had a significant positive linear effect ($\beta =0.12$, $z=4.90$, $p<.001$) and a significant negative squared effect ($\beta =-0.06$, $z=-3.41$, $p<.001$). Together, they accounted for the inverse U-shape. The magnitude of point values had a significant positive effect on engagement ($\beta =0.08$, $z=6.05$, $p<.001$), as was predicted (see Fig. 4f).

## Discussion

We studied the influence of difficulty on intrinsic motivation — with a particular focus on the learning progress theory — in inherently enjoyable environments, based on three large-scale game data sets and two controlled experiments. Across three video games (a puzzle, a racing, and a platformer game, see Fig. 2), we found that player’s ratings depended on the difficulty of the level, as predicted by a range of inverted-U shape theories. From a normative perspective, intermediate difficulty led to the most learning progress, which in turn led to higher ratings (indicating increased fun), which we were able to test in naturally enjoyable and engaging game settings. In the Super Mario Maker data set, we observed two additional factors that also influenced ratings: i) the difficulty-expectation disparity between prior expectations of difficulty and our difficulty calculations based on the success ratio of the task and ii) the effect of success in easy levels. Through two precisely controlled experiments — a guessing game (see Fig. 3) and a grid exploration game (see Fig. 4) — we confirmed that both of these additional components influenced player’s engagement beyond the influence of learning progress.

As a first factor, we found an influence of a difficulty-expectations disparity: Players’ engagement was maximized in environments with a difficulty that matched their prior expectations of difficulty — indicating that they like to reduce their prediction error as fast as they predicted. This is in contrast to previous theories of fun and enjoyment, which state that fun is maximal when players reduce their prediction error faster than expected^46,47^ — indicating that players should most enjoy acting in environments that are less difficult than expected.

As a second factor, we found an influence of success: Players’ engagement was maximized in environments with higher underlying point values. This aligns with findings from earlier studies that showed that different kinds of game rewards can influence enjoyment^39^. Previous work already suggested that players enjoy a feeling of performing well, which we believe occurred by finding high point values^48^.

These results emphasize the benefits of combining naturalistic large-scale data sets with controlled experiments. Through this usage of a diverse set of paradigms and measurements, we were not only able to confirm the influence of difficulty on intrinsic motivation, but also discover additional factors relevant for players’ experience.

### Limitations and future directions

We start this paper by arguing that we want to understand people’s experience of “fun” or “enjoyment”. However, the exact nature of these concepts has been debated^31,32^. For example, Blythe and Hassenzahl^32^ define “fun” as a subset of enjoyment and contrast it with “pleasure”. They argue that fun is more trivial and short-lived, while pleasure is concerned with relevance and identity. Other work in psychology use the term pleasure more generally for enjoyable experiences^49^, while other work describe similar concepts as “momentary subjective well-being” or “momentary happiness”^50^. In their work on learning progress, Oudeyer et al. use the term “intrinsic reward”^16^ as the dependent variable in their theory. This is linked to the literature on intrinsic motivation, which suggests that certain properties of environments, such as novelty or surprise, can be intrinsically rewarding for agents^16,51^. In our work, we aim to describe and understand the positive affect associated with intrinsically motivating paradigms, such as games. To do so, we use the terms “enjoyment” or “intrinsic reward” as appropriate. Thus, while Schmidhuber uses the expression “fun”, we do not claim this to be the only appropriate term to refer to the researched concept.

In Experiment 2, we showed that participants were motivated by the magnitude of point values. Whether these point values can be categorized as intrinsic rewards is debatable. On the one side, the concept of “more points are better” is learned by the participants in many natural environments, which speaks for the categorization as extrinsic reward. However, in many computer games the points do not have any direct consequences on the gameplay. In our experiment, we even went a step further and did not accumulate the points and additionally instructed players that they would not be compensated for them. This speaks in favor of a categorization as intrinsic reward. Because of similar difficulties in other environments, it has been argued that rewards might lie on a spectrum between extrinsic and intrinsic^52,53^. In this framework, our instantiation of point values might lie between the two extremes of purely intrinsic and extrinsic rewards. Other researchers even argue whether categorizing rewards into the dimension of intrinsic-extrinsic is sensible at all and propose to instead look at the specific properties of the different rewards^54^.

While the influence of task difficulty on intrinsic motivation — as assessed in our game datasets — is consistent with multiple theories predicting an inverted-U relationship^4^, our experiments specifically focus on the theory of learning progress. As previously noted, many of these theories converge on similar predictions regarding difficulty and can be integrated into a broader framework of knowledge maximization^4^. Among them, the theory of learning progress offers a normative account of intrinsically motivated behavior. We believe that — even in activities pursued purely for fun — people are motivated by the desire to make learning progress and tend to seek out environments that maximize it.

In this current work, we were only able to examine learning progress through simulations since it could not be directly assessed in our real-world games dataset, as we do not have access to the individual histories of each player. It has been shown that people can monitor their learning progress accurately^25^, but it is still an open question for which specific tasks this is possible. For instance, it might be the case that monitoring learning progress in tasks characterized by slow scales of learning such as in motor-skill learning poses a challenge for humans^27^, and different heuristics are available in different settings^55^.

However, we still believe that the higher ratings and increased engagement with intermediate difficult stimuli in our data stems from learning progress, since the qualitative results of the learning progress simulations point to similar preferences. In our guessing game, we simulated learning progress using a Kalman filter, which stopped sampling when the estimated mean of the distribution converged and subsequent updates become negligible. The results of the simulation revealed a similar pattern to participants’ preferences. Likewise, in our grid exploration task, simulating player behavior by iteratively updating a model of the grid’s underlying structure produced qualitatively similar results to those observed in the human data. Furthermore, it has been shown that a preference for intermediately difficult stimuli disappears if the sense of learning progress is suppressed^29^. Thus, the preference for intermediately difficult environments might not stem from a conscious decision to maximize learning progress, but rather an intrinsic feeling of enjoyment as we have investigated here.

And although the theory of learning progress offers a normative account of intrinsic motivation, the underlying cognitive mechanisms still remain unclear^4^. Ten et al.^4^ argue that learning progress is just one of several theoretical perspectives, each highlighting different factors — such as progress^13^, uncertainty^21,56^, expectancy^2^ or familiarity^57^ — that may drive intrinsically motivated behaviour. To disentangle these influences and better understand the origins of people’s preferences, new experimental paradigms capable of distinguishing between these factors are required.

While our results and previous theories suggest that people enjoy interacting with environments in which they can maximize knowledge, people can find enjoyment in situations where this is not the case. On the one hand, people sometimes enjoy tasks where no learning progress is possible, such as repeatedly solving a Rubik’s Cube when already knowing how to do it, or interacting with nearly impossible tasks^58^. On the other hand, people might make progress in learning, but still not enjoy the activity, such as studying for a boring exam. Instead of trying to accommodate every situation in which learning progress could matter, the goal of the current work was to closely investigate the inverted U-shape theories like learning progress in realistic settings to capture the main aspects of fun while identifying additional concepts influencing enjoyment.

Arguably, the main motivation for playing computer games is that they are fun and provide an enjoyable experience. While different levels of a game can be considered more or less enjoyable, they all represent potentially fun tasks. In our controlled experiments, we aimed to cover a broader range of experiences, including rather boring conditions. However, we ensured that participants interacted with the task only if they were motivated, by instructing them that compensation was entirely independent of their performance or level of engagement. We therefore assume that, despite the simplicity of our paradigms, participants’ engagement is at least partly driven by intrinsic motivation. In the future, we plan to develop more complex paradigms that are inherently more enjoyable while still allowing for a high level of experimental control.

In our work, we approximated the positive affect of “enjoyment” using two measures: the amount of engagement with the experiment and players’ ratings. We believe that using a diverse set of measurements is beneficial for a comprehensive understanding of human experiences. However, these two measures capture distinct theoretical constructs. While engagement reflects persistence and effort (“wanting”), level ratings represent a retrospective evaluation of the experience (“liking”)^59^. These constructs can lead to different predictions about human behavior. Though traditionally considered two sides of the same coin, the distinction between “liking” and “wanting” is now well established in neuroscience, referring to distinct psychological and neurological processes^60^. In our work, we adopt a multimodal approach to studying the higher-level concept of enjoyment in paradigms aimed at intrinsic motivation. Both engagement and subjective ratings have been widely used in intrinsic motivation research (e.g.^3,7^) and, more specifically, in studies on learning progress^25,61^. By integrating these two sources of evidence — both encompassed by the broader concept of “enjoyment” — we aim to deepen our understanding of this phenomenon. In the future, the influence of different intrinsically motivating components on distinct psychological constructs, such as “liking” vs. “wanting” should be further disentangled. This can be achieved by incorporating multiple measurements within the same paradigm^4^, and by capturing enjoyment more explicitly — such as through direct self-reports or psychophysiological measures like facial expressions and heart rate variability^62,63^.

We simulated players as passively confronted with tasks of unknown difficulty. However, in video games, as well as in real life, people can often decide on the difficulty of the next task, or follow a predefined curriculum with increasing difficulty. While previous studies have demonstrated that learning progress plays a role in actively choosing difficulties^25,27,61^, there exist situations in both video gaming and real life where people may not know how difficult a task is going to be or may only have vague prior intuitions. With many unexplored options available, instead of actively choosing the next task based on an estimate of difficulty, people often encounter new tasks about which they do not know anything: For example, when attempting to write a paper or trying out a new sport, one might not necessarily know how difficult it will be, yet can still experience enjoyment. However, people nonetheless need to assess whether and how long to engage in these tasks. Therefore, we think that our experiments capture important aspects of everyday activities, as participants also do not know the difficulty of a task beforehand, but still need to decide whether to continue engaging in it.

In addition to the difficulty level, players in the three game datasets have access to various pieces of information about each level before deciding which one to engage with next. As a result, their choices may be influenced by a range of other psychological factors — for example, individual differences in preferences and the extent to which they are affected by social cues^64^, such as how much other players liked a particular level^65^. While participants could not choose the levels they were confronted with in our experiments, thereby remedying this potential confound, future research should aim to disentangle the different factors that people consider when selecting between multiple intrinsically motivated activities in richer settings.

While it is important to identify the set of possible strategies used by humans in non-instrumental settings, future research should further explore when and where people rely on which factor influencing their enjoyment, and how different factors might interact^26,66,67^. For example, work by Dubey and Griffiths^6^ reconciled novelty-based and complexity-theories, by showing that their usefulness depends on the structure of the environment and that people use them accordingly. Although the rich environments here allowed us to uncover two factors influencing enjoyment beyond the inverted-U relationship, we can currently not examine the combined influence of the difficulty-expectation disparity and the feeling of success (induced by point values), as well as how much people might rely on one or the other. Thus, a more detailed analysis of how different factors interact will require the development of new games that provide access to individual skill levels and preferences, a thorough manipulation of prior expectations of difficulty, and a careful control of point values. Further research focusing on the interaction between these factors will be key to advancing our theory of human behavior in non-instrumental settings.

### Conclusion

We investigated the relationship between difficulty and intrinsic reward in three richly structured, large-scale game data sets and two simple experiments, which focus on enjoyment, rather than learning. We found that — as predicted by multiple inverted U-shape theories, such as learning progress — players preferred environments of intermediate difficulty. On top of that, we found in one of the game data sets, as well as in two highly-controlled experiments and detailed simulations, that a difficulty-expectation disparity, as well as a sense of success had additionally influenced enjoyment. These results enrich our understanding of the dynamics of fun in realistic environments and emphasize the importance of using both realistic, game-like environments and highly controlled experiments together with detailed simulations to advance theories of human learning and decision-making.

### Materials and methods

The guessing game experiment was approved by the ethics committee of the medical faculty at the University of Tübingen (number 701/2020BO). The grid exploration game was approved by University College London Ethics Board. Both experiments were carried out in accordance with the relevant guidelines and regulations and informed consent was obtained from all subjects before participation. Both experiments were created with standard JavaScript. The simulations and data analyses were conducted in R and Python.

#### Game data sets

The Robozzle data set was created from the information on the official website of the game (http://robozzle.com/js/index.aspx^68^), which lists all generated levels. The data set includes all data from the “puzzle list” available in October 2020.

The Trackmania data set was created from information available on the webpage “Trackmania Exchange” (https://tmnf.exchange/^69^). We included all generated race tracks available in November 2020.

The Super Mario Maker data set was provided on kaggle (https://www.kaggle.com/datasets/leomauro/smmnet/data^70^). Its data originates from SMM Bookmark, the official website of Super Mario Maker, which was discontinued in March 2021. The data from all three data sets is publicly available and does not include any identifiable information.

#### Guessing Game

Players of the guessing game were told that they were scientists on an intergalactic mission, who stopped on an alien planet to get their spaceship fueled. They needed to pass ten minutes on an alien playground until the spaceship would be ready. On this playground, they found machines that produced numbers according to different Gaussian distributions. Each machine sampled from a Gaussian distribution with a fixed mean — uniformly sampled between 20 and 80 — and a fixed variance — uniformly sampled out of the following five values: 0.1, 1, 10, 100, 1000. Players always interacted with one machine at a time. They could guess the next number (between 0 and 100), the current machine would produce — if the machine would generate a number that lies below 1 or above 100 it would resample. After participants submitted their current guess through interacting with a slider, the machine displayed the number it produced, as well as the difference between this number and the participant’s guess. After each guess, players could decide whether they wanted to make another guess or go on to the next machine, which would have a new fixed mean and variance (with the constraint of having to guess at least three times per machine before going on). The order of the machines participants encountered was randomized. Participants were not able to visit a machine again, once they advanced to the next one. Players were instructed that the compensation consists of 3$, with no option for additional compensation. The instructions also made clear that the study would end after 10 minutes independent of their engagement — with how many machines they played or how many guesses they made — or of their performance. The experiment automatically ended when the participant pressed a button once the time was up. Therefore, some participants did not encounter all 5 different variances, as they only visited a few machines.

We recruited 103 participants on Amazon’s Mechanical Turk (30 females, mean age 33.26, SD 9.65). We excluded 4 participants, as they needed at least 10 retries on our comprehension check, consisting of four multiple-choice questions. Additionally, we removed the data on the last machine of every participant, as we could not assess how long they would have liked to interact with that machine. Therefore, we removed one additional participant, who played with only one machine. In the end, we used the data of 98 participants for our analyses. Participants played on average with 14 machines (mean 13.68, SD 8.55), made on average 77 guesses in total (mean 77.35, SD 27.65) and 8 guesses per machine (mean 8.42, SD 7.74).

#### Grid exploration game

Players of the grid exploration game were told that they would explore different grids with 30x30 tiles, with values from 5 to 75. They encountered one grid at a time, iteratively opened its tiles and observed their point values together with a corresponding color. At each time point, players could decide which new tile they want to open or to go on to the next grid (with the constraint of having to open at least 5 tiles per grid). When encountering a new grid, only one tile was revealed. They were not able to go to a previously encountered grid. Participants did not receive any specific goal, instead they were told to explore the grids. They were instructed that the experiment would automatically end after 10 minutes, no matter how many grids they interacted with or how many tiles they opened. Once participants left a grid after 10 minutes were over, the task ended. They also were instructed that they are compensated with 3$ independent of their performance or engagement in the task.

The grids were generated using a Gaussian process with a radial basis function kernel. We manipulated the smoothness of the grids, by uniformly sampling the length-scale parameter ($\lambda$) of the kernel from the following values: 0.25, 0.5, 1, 2, 4, 8, 16. The tile values of each grid spanned a range of 40 values. For each grid, we uniformly sampled a value between 5 and 35, as the lowest value a tile would display, thereby setting the highest value between 45 and 75. The value of each tile was visualized by a shade of red. Low values had a lighter shade, while high values had a darker shade. Every time a participant clicked on the button “Next”, they encountered a new grid with a new smoothness and new range of values. Each grid was randomly sampled at the time of advancing, therefore the order of grids was different between subjects. Therefore, some participants did not experience all length-scale values (dependent on the number of grids they visited).

We recruited 44 participants on Amazon Mechanical Turk (18 females, mean age 31.1, SD 6.84). In this experiment, we did not exclude any participant from our analysis. Participants interacted on average with 40 grids (mean 39.57, SD 37.16) and opened on average 36 tiles per grid (mean 36.09, SD 28.99).

## Supplementary Information

Supplementary Information.

## Data Availability

Anonymized participant and player data, as well as the code used for the experiments, simulations, and analyses are available at https://github.com/franziskabraendle/fun_learning_progress..
