# Supplementary material for: Leveling up fun: learning progress, expectations, and success influence enjoyment in video games
Source: Sci Rep. 2025 Oct 1;15:34153. doi: 10.1038/s41598-025-14628-2 (PMC12488971; doi:10.1038/s41598-025-14628-2)
Supplement: Supplementary file 1 — Supplementary Information. [file 41598_2025_14628_MOESM1_ESM.pdf]

# Supporting Information

## Game data sets

### *Data cleaning*

We cleaned the game datasets before using them for our analyses. As we had access to very different formats of data in the three games, we applied different methods for cleaning the respective data. In Robozzle, we excluded one level including null values in the difficulty and rating, and all levels that had a “?” instead of a value as their difficulty (66 of 8241). Additionally, we excluded levels that had no ratings – neither likes nor dislikes (180 of 8241). Thereby, we reduced the data set from 8241 to 7994 levels.

In Trackmania, we included all 128662 levels, as every level had at least one award.

In Super Mario Maker, several levels were included in the dataset multiple times, with the data extracted from the game website at different time points, spanning several months. For our analysis, we only included the data of the newest time point per level. We then included all 115032 levels in our analysis, even if they did not have any rating (here called star). We think it is reasonable to include levels without any rating, as – in contrast to Robozzle – players cannot give negative ratings, and no ratings might just be a sign of players not liking the level. All the levels were attempted by at least 10 players. We believe that this might have been a pre-selection by the creator of the data set.

### *Data visualization*

To give an insight into the distribution of difficulty and rating values, we plotted the data for all three datasets together with their marginal histograms (see Fig. 1).

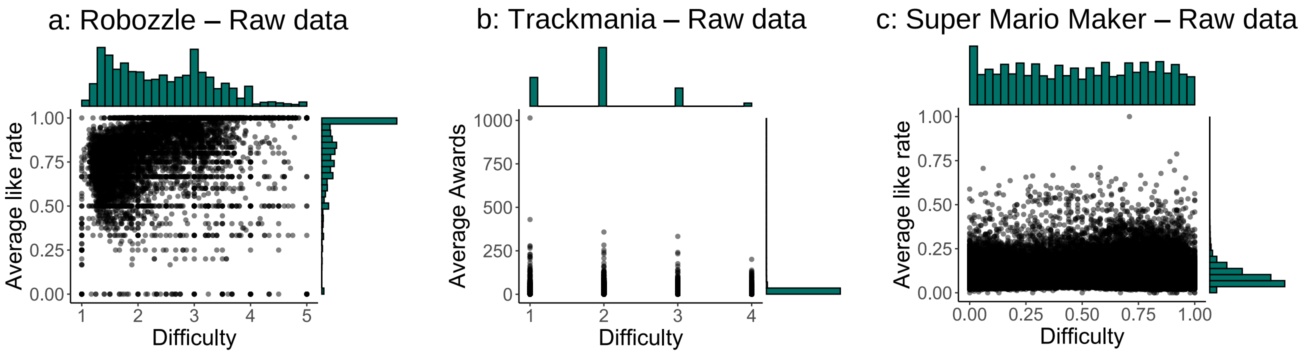


**Figure 1.** Raw data of human rating behavior in game datasets, including marginal histograms **a:** Raw data of people’s ratings of different levels of Robozzle. **b:** Raw data of people’s ratings of different levels of Trackmania. **c:** Raw data of people’s ratings of different levels of Super Mario Maker.

### *Super Mario Maker – Categories*

In the analysis of the Super Mario Maker data set we used the given categories to approximate participants’ expectations. When plotting the relation between the players’ ratings and the true difficulty for the individual categories, we found that the given difficulty categories do often not correspond to the true difficulty, but span almost the whole range of difficulty values (see Fig. 2). This indicates that the label of the difficulty category might be misleading for many levels.

### *Model Comparison*

Due to the size of the three datasets, standard null-hypothesis testing methods yield trivially significant results for increasingly higher-degree polynomials, despite only modest improvements in fit. Therefore, we used a scree plot to identify the “elbow” where the goodness of fit begins to level off with increasing model complexity. Specifically, we measured goodness of fit with the mean squared error of the residuals (see Fig. 3), and analyzed how they improved for polynomial degrees 1 through 10 for each dataset. We observed that adding a quadratic term meaningfully improved model fit for all three datasets. However, in the Super Mario Maker dataset, adding a cubic term did further reduce the mean squared error noticeably, while a fourth-degree polynomial did not. This aligns with our observation that ratings tend to have a negative relationship with difficulty in the simplest levels, while they follow an inverted-U relationship in levels of higher difficulty.

### *Super Mario Maker – Analysis of difference between calculated difficulty and expected difficulty*

In our analysis of the difference between the categories and our calculated difficulty measure, we mapped the four category levels to the average calculated difficulty of the levels they include – very easy: 0.15, normal: 0.47, expert: 0.79, super expert:

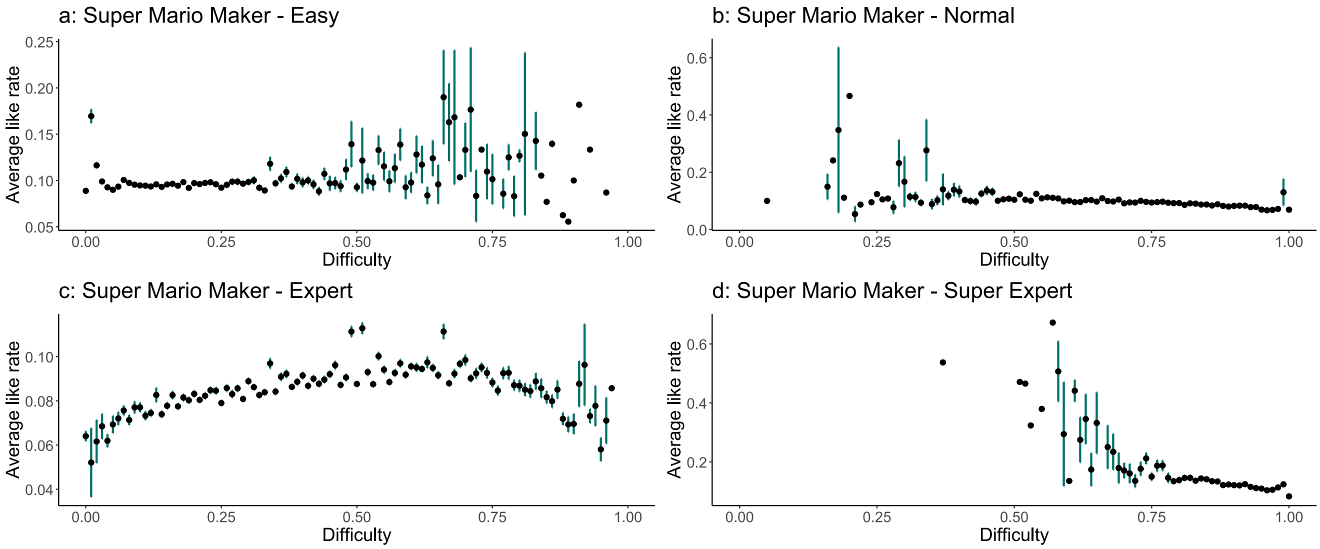


**Figure 2.** Human rating behavior in the Super Mario Maker game dataset, divided by given difficulty category. We used different y-axis for the four plots to improve visibility of the different trends. **a:** People's ratings of different true difficulty levels, labeled as "easy". **b:** People's ratings of different true difficulty levels, labeled as "normal". **c:** People's ratings of different true difficulty levels, labeled as "expert". **d:** People's ratings of different true difficulty levels, labeled as "super expert". Error bars indicate the standard error of the mean.

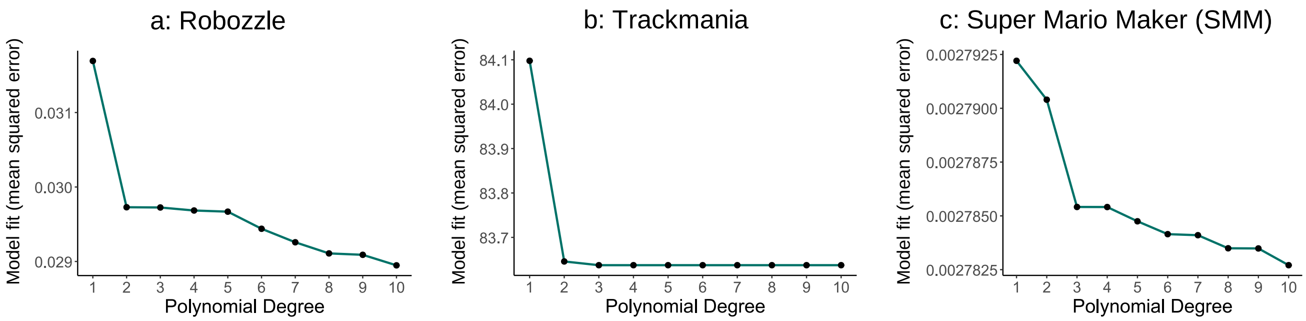


**Figure 3.** Goodness of fit as a function of polynomial degree for the linear regression models. Goodness of fit is measured by the mean squared error of the residuals from polynomial regression models with degrees ranging from 1 to 10. **a:** Goodness of fit for Robozzle. **b:** Goodness of fit for Trackmania. **c:** Goodness of fit for Super Mario Maker.

0.92. We then took the difference between the calculated difficulty of each level and this prior expected difficulty according to the category the level is placed in. In our regression analysis, we included a linear and quadratic component of this difference. For our main analysis, we did not scale the difference, to be able to directly interpret the x-value of the peak of the inverted U-shape. However, we still saw a significant negative effect of the quadratic component and only a very small effect of the linear component, when scaling the difference (linear: $\beta = 0.00$, $z = 6.72$, $p < .001$, quadratic: $\beta = -0.01$, $z = -40.12$, $p < .001$), as in the original analysis.

## Guessing game

## Summary statistics

We checked whether the manipulation of variances indeed had an influence on the difficulty of guessing the correct number. We report the average difference between participant's guesses and the numbers produced by the machine, averaged per participant and variance in Table 1. Additionally, we report the number of correct guesses and ratio of correct guesses per variance across all participants. For these analyses, we excluded the first guess made for each machine, as it is by definition always random. We found a significant effect in a regression analysis between participant's guesses and the scaled base-10 logarithm of the variance and found a ($\beta = 5.79$, $t = 33.03$, $p < .001$). We also found a significant correlation between the variance and the number of correct guesses ($r = -0.94$, $p = .016$).

**Table 1.** Summary statistics of the guessing performance.

| Variance | Average difference of guesses | Total nr. of correct guesses | Ratio of correct guesses |
|---:|---:|---:|---:|
| 0.1 | 7.57 | 282 | 0.28 |
| 1 | 8.85 | 247 | 0.17 |
| 10 | 10.03 | 73 | 0.05 |
| 100 | 13.84 | 36 | 0.03 |
| 1000 | 25.14 | 17 | 0.01 |

### *Main regression analysis*

For the main regression analysis of the guessing game, we used a negative binomial mixed effects model. We took the base-10 logarithm of the variance – as its values range from 0.1 to 1000 –, but did not scale it. This has the advantage that by including the beta coefficients into a quadratic equation, the peak and thereby the preferred variance of players is directly interpretable. Nevertheless, when scaling the variance before including it in the regression analysis, the squared variance still had a significant negative effect. Additionally, we included random slopes for each participant to the analysis. We still see similar results as before when not scaling the values. The model did not converge when scaling the values. The regression results of the different analyses can be found in Table 2.

**Table 2.** Regression coefficients for the mixed effects analysis of the guessing game.

Main analysis

| | Estimate | Std. Error | z value | Pr(>\|z\|) |
|---:|---:|---:|---:|---:|
| Variance | 0.07 | 0.02 | 2.99 | 0.00 |
| Variance squared | -0.03 | 0.01 | -3.40 | 0.00 |

Using scaled variance

| | Estimate | Std. Error | z value | Pr(>\|z\|) |
|---:|---:|---:|---:|---:|
| Variance | 0.00 | 0.02 | -0.02 | 0.99 |
| Variance squared | -0.07 | 0.02 | -3.40 | 0.00 |

Including random slopes

| | Estimate | Std. Error | z value | Pr(>\|z\|) |
|---:|---:|---:|---:|---:|
| Variance | 0.00 | 0.02 | -0.02 | 0.99 |
| Variance squared | -0.07 | 0.02 | -3.40 | 0.00 |

### Individual data

We, additionally, looked at the individual coefficients per player for our main regression analysis, including random intercepts and slopes. We saw that for 76 of the 98 analyzed players, the coefficient of the variance squared was negative, as predicted by the theory. For 64 of these, the peak of the inverted U-shape was between the base-10 logarithm of 0.1 and 1000 indicating that they actually exhibit an inverted U-shaped behavior within the tested variances.

### Different versions of data cleaning

For the main guessing game analysis, we only excluded participants who needed at least ten trials for the comprehension check. We decided to not exclude any additional participants, as we wanted to assess participants' behavior when acting according to their enjoyment, as they were instructed to, which meant that they were allowed to do whatever they wanted.

However, we still wanted to analyze whether the exclusion of participants creating a low amount of data changed our results. Therefore, we tried out two additional methods of cleaning the data: excluding all participants who never made more than 3 guesses per machine and participants who did not play with more than two different variances. Both of these groups would not display an inverted U-shape in their behavior. We saw that when excluding both of these groups – thereby reducing the data set to 83 participants, we still saw a significantly negative squared effect of the variance, as expected (see Table 3).

**Table 3.** Regression coefficients for the mixed effects analysis of the guessing game, excluding participants who never played more than three guesses and who interacted with only two different variances.

| | Estimate | Std. Error | z value | Pr(>\|z\|) |
|---|---|---|---|---|
| Variance | 0.08 | 0.03 | 3.08 | 0.00 |
| Variance squared | -0.03 | 0.01 | -3.26 | 0.00 |

### Analysis of expectations

We analyzed the expectations of participants by including their previously observed machines into our model. This was done by first estimating the true underlying variance for each machine a participant encountered, based on all the generated numbers the participant observed before continuing to the next machine. We then averaged for each machine the estimated true variances from all the machines encountered before, weighted by number of guesses participants played with each. This was used as an approximation of players prior expectation of the variance of the current machine. We then took the difference of the base-10 logarithm of the estimated true variance and the estimated expected variance. We included the scaled difference in a negative binomial mixed-effects regression analysis, together with the scaled number of machines the participant has seen so far. With this setup, we found an inverted U-shape relationship, as expected. We changed some details of the procedure – not scaling the difference, not weighing the machines to create the expected variance, taking the base-10 logarithm before averaging to calculate the expected variance – to see whether the results were robust. In all cases, we saw a significant negative effect of the squared variance, as expected. As the influence of the linear variance component was never significant, the peak of the inverted U-shape always was at a difference of 0 between the expected and the true difficulty. The details of the different regression analyses can be found in Table 4.

### Rating data

We approximated players' enjoyment by the number of guesses they played with different machines. As an additional measure, we asked players after each machine how much fun they were having on a scale from 1 - 7. We did not include the results of this questionnaire, as we believe that this is not the ideal way to assess enjoyment in an experiment in which players can decide how long to engage with each task. As we only asked players after interacting with a machine, they might already have played with it for many trials, and the enjoyment at the current moment might be different from the enjoyment at the beginning.

### Simulation and threshold

We simulated the guessing game by using a Kalman filter. We set the prior mean to 50 (the average of the possible value span), and the prior variance to one of the five considered variance values – 0.1, 1, 10, 100, 1000. We then calculated the Kalman Gain, the updated mean, and the updated variance according to the following equations:

$$K_t = \frac{\hat{v}_{t-1}}{\hat{v}_{t-1} + v_{\text{true}}}$$

$$\hat{\mu}_t = \hat{\mu}_{t-1} + K_t \cdot (x - \hat{\mu}_{t-1})$$

**Table 4.** Regression coefficients for the mixed effects analysis of the guessing game including expectations

Main analysis

| | Estimate | Std. Error | z value | Pr(>\|z\|) |
|---|---|---|---|---|
| Variance | 0.02 | 0.02 | 1.33 | 0.19 |
| Variance squared | -0.06 | 0.01 | -4.16 | 0.00 |
| Machines | -0.06 | 0.02 | -2.67 | 0.01 |

Without scaling the difference

| | Estimate | Std. Error | z value | Pr(>\|z\|) |
|---|---|---|---|---|
| Variance | -0.02 | 0.01 | -1.73 | 0.08 |
| Variance squared | -0.02 | 0.00 | -4.16 | 0.00 |
| Machines | -0.06 | 0.02 | -2.67 | 0.01 |

Without weighting the previous machines according to the number of samples

| | Estimate | Std. Error | z value | Pr(>\|z\|) |
|---|---|---|---|---|
| Variance | 0.02 | 0.02 | 1.35 | 0.18 |
| Variance squared | -0.04 | 0.01 | -3.29 | 0.00 |
| Machines | -0.06 | 0.02 | -2.56 | 0.01 |

Taking the base-10 logarithm before taking the average to calculate the expected variance

| | Estimate | Std. Error | z value | Pr(>\|z\|) |
|---|---|---|---|---|
| Variance | 0.02 | 0.02 | 1.20 | 0.23 |
| Variance squared | -0.06 | 0.01 | -4.29 | 0.00 |
| Machines | -0.06 | 0.02 | -2.76 | 0.01 |

$$\hat{v}_t = \hat{v}_{t-1} \cdot (1 - K_t)$$

$K_t$ is the Kalman Gain used to update the mean and variance estimates based on the observed sample $x$ and the true variance $v_{\text{true}}$. $\hat{\mu}_t$ represents the estimated mean, $\hat{v}_t$ represents the estimated variance or uncertainty over the mean.

We simulated 1000 runs per variance for each prior variance. During each run, we defined the distribution for the current machine by taking a fixed mean – uniformly sampled between 20 and 80 – and a fixed variance as defined by the current run. At each time step, the distribution returned a sampled value between 1 and 100 (resampling if the value was outside that range). We stopped a run if the absolute difference between the last and the current estimated mean fell below a certain threshold. As in the main experiment, the simulation needed to sample each machine at least three times. In our main analysis, we set the threshold arbitrarily to 1. However, as can be seen in Figure 4, changing the threshold does not change the qualitative predictions of the simulation.

## Exploration game

### *Grid design*

We created different grids for the exploration game by using a Gaussian process with an underlying radial basis function kernel. By manipulating the lengthscale value ($\lambda$) of the kernel, we created more or less smooth environments. We decided on the values 0.25, 0.5, 1, 2, 4, 16, as these provide a range of grids with a variation of spatial correlations and thereby with different smoothness (see Fig. 6). Additionally, we manipulated the magnitude of point values of each grid. While each grid has a range of 40 point values, we uniformly sampled the lowest value of each grid to lie between 5 and 35 (and thereby the highest value between 45 and 75). We additionally visualized the exact point values with a shade of red – the higher the value the darker the red (for examples of grids with different smoothness and magnitude, see Fig. 5).

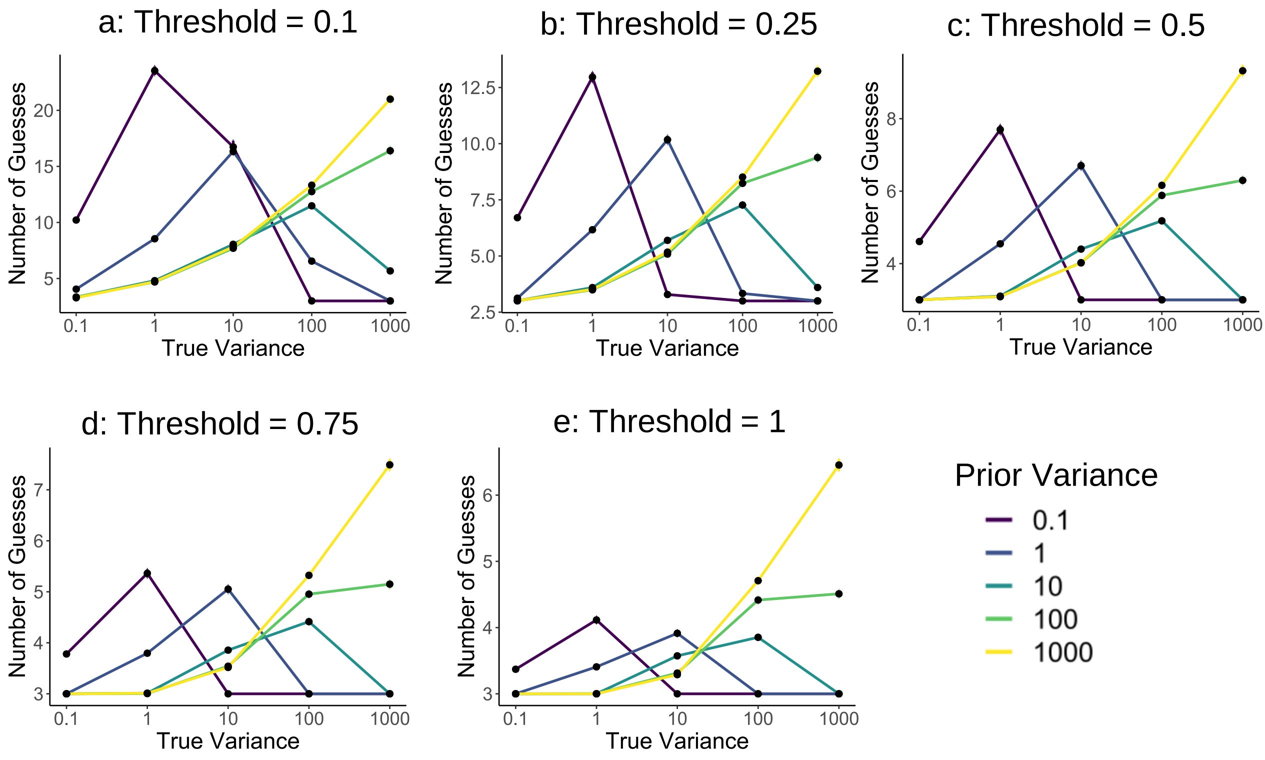


**Figure 4.** Simulation of the guessing game for different prior variances. **a - e:** Simulation results for different thresholds ranging from 0.1 to 1. The higher the threshold, the sooner the simulation stops – i.e. after fewer samples. The shapes of the different curves are qualitatively similar, regardless of the threshold used.

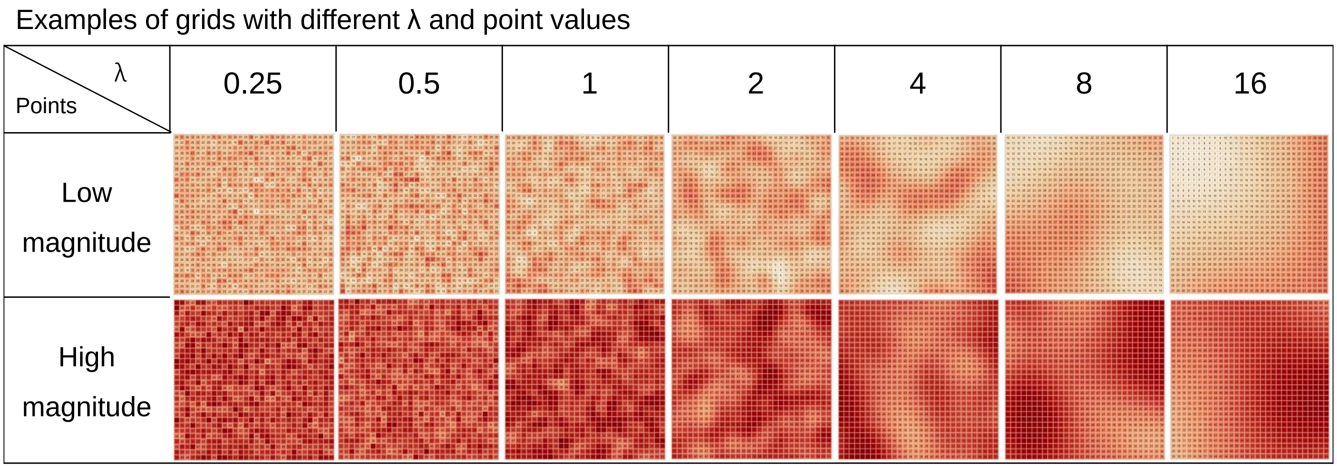


**Figure 5.** Examples of generated grids. We generated grids with a radial basis function kernel with seven different $\lambda$ values. These influence the roughness and smoothness of the grid. We also manipulate the magnitude of point values of the grids, indicated by the color of the tiles.

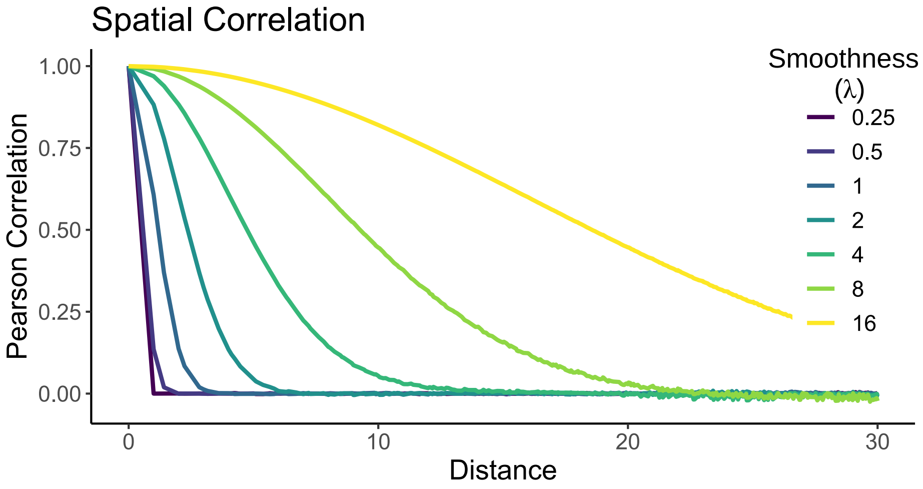


**Figure 6.** Spatial correlation between the values of tiles, dependent on the distance, plotted for the seven different $\lambda$ values. The higher the $\lambda$ value, the stronger the correlation.

### *Main regression analysis*

For the main regression analysis, we again used a negative binomial mixed effects model. We took the smoothness ($\lambda$) – its values range from 0.25 to 16 – directly without base-2 logarithm, and the magnitude – its values range from 45 to 75. Additionally, in contrast to the guessing game, we directly scaled the values, as not scaling them led the model to not converge (see Table 4). We again tried to include random slopes for each participant, however, the model showed a singular fit. Nevertheless, we report it in the following table, as we used these values for the analysis of the individual data (see Table 5).

Additionally, we tried including an interaction effect between the lengthscale ($\lambda$) and the magnitude. We found a significant interaction effect between the magnitude and the linear $\lambda$ component (see Table 5). However, when looking at the model comparison, the interaction effect did not improve the model (original model: BIC 11752, model with interaction effect: BIC 11762).

### *Individual data*

We, again, looked at the individual coefficients per player in our main regression analysis, including random intercepts and slopes. As reported in the paragraph above, this model exhibited a singular fit, so should be interpreted with care. Nevertheless, we used this analysis to inspect the individual slopes of participants as before in the guessing game. Out of the 44 participants, all 44 had a negative squared coefficient of $\lambda$. For 41 out of these, the peak of the predicted inverted U-shape occurred within the range of the tested $\lambda$ values. This again shows that most participants actually exhibited an inverted-U shaped behavior. Additionally, 37 out of the 44 participants showed a positive linear effect of the magnitude of point values.

### *Simulation and threshold*

We simulated the grid exploration game by using a Gaussian process model with a radial basis function kernel. For each lengthscale parameter ($\lambda$), we created 1000 grids. We then simulated an agent iteratively sampling tiles of the current grid in a random fashion. After each sample, the Gaussian process model is updated and optimized – it adapts its $\lambda$ value to the current environment. The new model then makes predictions over the whole grid. We compared these predictions at each step to the ground truth – the underlying values of the grid – and calculated the total mean squared error. Again, as in the guessing game, the agent continued sampling until it did not significantly update its predictions anymore. We implemented this by comparing the error at the current time step with the error of the previous time step. If this error lay below a predefined threshold, the agent would stop interacting with the current grid. In our main analysis, we set the threshold again to 0.5 for simplicity. As in our experiment, the simulation had to sample at least five tiles. However, the results of the simulation look qualitatively similar, even when using different thresholds – the agent just sampled more or less trials (see Fig. 7).

**Table 5.** Regression coefficients for the mixed effects analysis of the exploration game

Main analysis

| | Estimate | Std. Error | z value | Pr(>\|z\|) |
|---|---|---|---|---|
| Variance | 0.12 | 0.03 | 4.90 | 0.00 |
| Variance squared | -0.06 | 0.02 | -3.41 | 0.00 |
| Magnitude | 0.08 | 0.01 | 6.05 | 0.00 |

Including random slopes (resulting in a singular fit)

| | Estimate | Std. Error | z value | Pr(>\|z\|) |
|---|---|---|---|---|
| Variance | 0.16 | 0.04 | 4.22 | 0.00 |
| Variance squared | -0.08 | 0.02 | -3.33 | 0.00 |
| Magnitude | 0.11 | 0.03 | 3.71 | 0.00 |

Including an interaction effect

| | Estimate | Std. Error | z value | Pr(>\|z\|) |
|---|---|---|---|---|
| Variance | 0.12 | 0.03 | 4.85 | 0.00 |
| Variance squared | -0.06 | 0.02 | -3.37 | 0.00 |
| Magnitude | 0.11 | 0.02 | 5.00 | 0.00 |
| Variance*Magnitude | 0.06 | 0.02 | 2.25 | 0.02 |
| Variance squared*Magnitude | -0.03 | 0.02 | -1.61 | 0.11 |

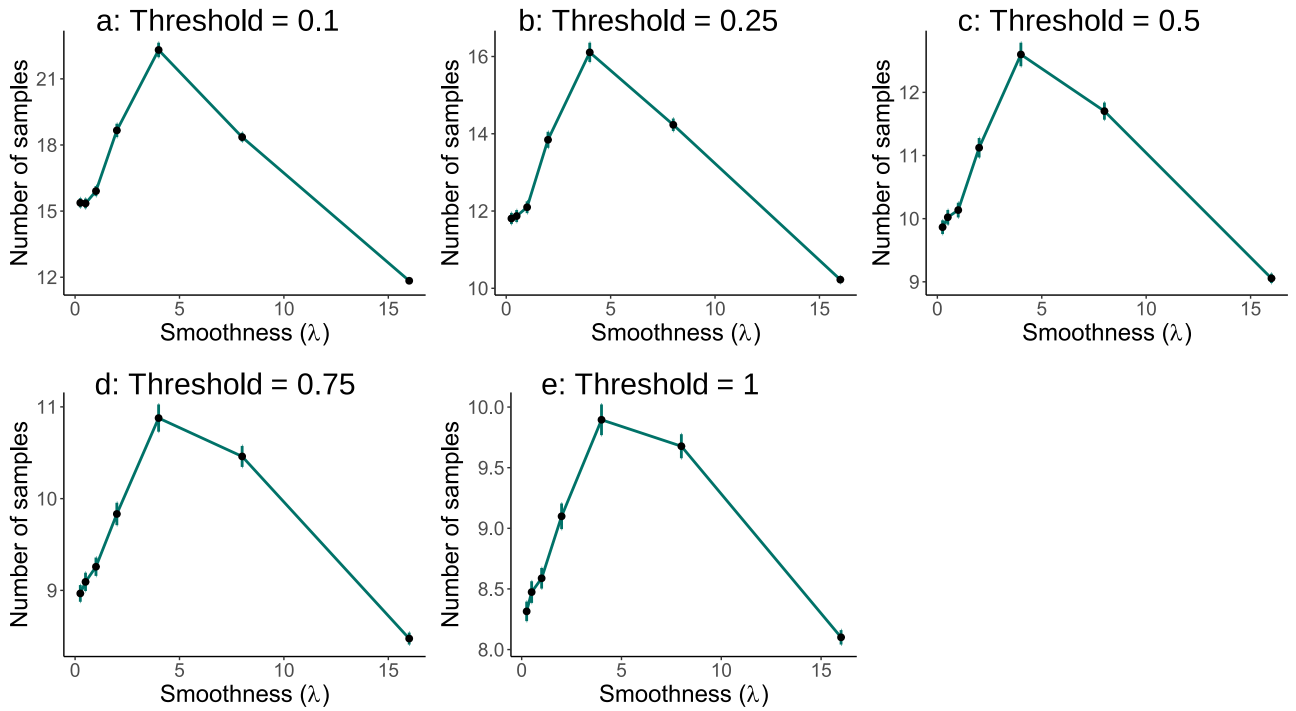


**Figure 7.** Simulation of the grid exploration task. **a** - **e:** Simulation results for different thresholds ranging from 0.1 to 1. The higher the threshold, the sooner the simulation stops – i.e. after fewer samples. The shapes of the different curves are qualitatively similar, regardless of the threshold used.
